# Radial Tunnel Syndrome Complicated by Lateral Epicondylitis in a Middle-Aged Female

**Published:** 2014-11-04

**Authors:** Sumesh Kaswan, Olivier Deigni, Kashyap K. Tadisina, Michael Totten, Bruce A. Kraemer

**Affiliations:** ^a^Division of Plastic Surgery, Saint Louis University, St Louis, MO; ^b^College of Medicine, University of Illinois at Chicago, Chicago, IL

**Keywords:** radial tunnel syndrome, radial tunnel release, posterior interosseous nerve, peripheral nerve entrapment, lateral epicondylitis

## DESCRIPTION

A 57-year-old right-handed woman presented to the plastic surgery clinic with a chronic history of left dorsal forearm pain over the extensor muscles with radiation to the hand, accompanied by numbness over the thumb-index web space. Pain was experienced during daily activities and was acutely worsened by resisted extension of the index and long fingers. She had a history of left lateral epicondylitis treated by her primary care physician and had had bilateral carpal tunnel releases. The patient's symptoms were managed unsuccessfully with anti-inflammatory medication and bracing. Surgical exploration yielded significant compression of the radial nerve at the arcade of Frohse, and a subsequent radial tunnel release using a dorsal approach was successfully performed.

## QUESTIONS

1. **What is radial tunnel syndrome (RTS) and what is the relevant associated anatomy?**
2. **What is the differential diagnosis in patients who have dorsal forearm pain?**
3. **How does one make the accurate diagnosis of RTS?**
4. **What are the treatment options for patients with RTS?**

## DISCUSSION

Radial tunnel syndrome, also referred to as radial pronator syndrome or posterior interosseus nerve entrapment, was first described in the year 1956. It refers to the entrapment of the deep branch of the radial nerve that causes significant pain and weakness (secondary to pain) for the patient. Because of nomenclature variation among the surgical community, this syndrome has also been referred to as posterious interosseus nerve (PIN) syndrome. This is because some surgeons refer to the deep branch of the radial nerve as the PIN when it is in the radial tunnel; although anatomically, the PIN is defined as the motor branch of the radial nerve once it passes under the superficial supinator muscle distally,^1^ and PIN syndrome is defined by true motor weakness.^2^ Radial tunnel syndrome can be caused by a variety of reasons, with the most common being trauma. Other causes include idiopathic entrapment, lipomas, cysts, or rheumatoid synovitis. Although incidence of this syndrome increases over time and has been attributed to repetitive use of the index and middle fingers during activities such as computer mouse use, currently, there are no significant data to support this notion. The radial tunnel is a space in the proximal forearm that extends from the radiocapitellar joint to the distal edge of the supinator muscle. It is bordered medially by the biceps and brachialis muscles, laterally by the brachioradialis, extensor carpi radialis longus, and extensor carpi radialis brevis (ECRB) muscles. The floor of the tunnel is composed of the head of the radiocapitellar joint through the deep head of the supinator muscle.^3^ The radial nerve splits at the elbow into the superficial radial nerve, which is a sensory nerve to the lateral forearm and dorsum of the hand, and the deep branch, which is a motor nerve innervating the ECRB and supinator muscles, as well as the other extensors of the wrist distally. A recent cadaveric study found that the deep branch of the radial nerve can be predicted to enter the proximal part of the supinator muscle at approximately 3.5 cm distal to the radial head and the PIN can be predicted to exit the distal part of the supinator muscle approximately 7.5 cm distal to the radial head.^1^ There are 5 main areas at which entrapment may occur within the radial tunnel: (1) fibrous fascia superficial to the head of the radius, (2) the recurrent radial artery and its associated veins (also called the leash of Henry) encircling the nerve, (3) the proximal border of the supinator muscle (known as the arcade of Frohse), (4) the distal border of the supinator muscle,^3^ and (5) the medial border of the ECRB muscle. The most common area of entrapment is the arcade of Frohse, followed by the leash of Henry, and the medial part of the ECRB muscle.^4^

Because of its mysterious nature, RTS is often confused with and sometimes presents with other compression syndromes, most commonly lateral epicondylitis, which has a much higher incidence (1%–3% annually) than RTS (0.003% annually).^5^ Radial tunnel syndrome was first discovered when physicians were unable to relieve discomfort in patients with lateral epicondylitis. These 2 syndromes may also exist concurrently, in which case they are often confused and misdiagnosed, with patients presenting with epicondylitis that can exacerbate symptoms of RTS.^6^^,^^7^ Furthermore, it is hypothesized that some cases of lateral epicondylitis that respond to conservative treatment can actually continue to have pain due to the associated development of RTS caused by fibrosis and scarring from the initial epicondylitis. Other conditions to be considered in the differential diagnosis include posterior interosseus nerve syndrome (entrapment distal to the radial tunnel resulting in true motor weakness), anconeus muscle tendonitis, supinator syndrome, brachial neuritis, deQuervains tenosynovitis, and simple tenosynovitis of the extensor tendons.^7^

The diagnosis of RTS is not without controversy. Because of rarity of the condition and the diagnosis being purely clinical with no established radiological or electrodiagnostic features, many surgeons and clinicians have denied its existence, challenged its diagnosis, and characterized symptoms as other related conditions. Incidence of RTS is estimated to be about 2.97 per 100,000 cases of radial neuropathy in men and 1.42 per 100,000 of new cases in women according to an epidemiological study of compressive neuropathies.^8^ In comparison, the rates of new cases in men/women for ulnar and median neuropathy were 24.4/18.1 and 82.0/198.9, respectively. Radiologically, magnetic resonance imaging has been used to evaluate RTS, and although some patients display signs of denervation edema or trophy within the extensor or supinator muscles, there is no statistical significance to these findings.^4^ Electrodiagnostic testing including nerve conduction tests, distal latencies, and electromyograms have been found to be unreliable when evaluating radial nerve compression, whereas these modalities have been found to be extremely useful when evaluating ulnar and median nerve pathology. Although these testing modalities can be used to rule out other causes of neuropathic pain, the diagnosis of RTS is completely clinically based. Patients have obvious tenderness over the radial tunnel distribution in the lateral dorsal forearm, about 3 to 5 cm distal to the lateral epicondyle over the supinator muscle, with associated weakness and a lack of sensory symptoms.^3^ However, muscle strength should be normal in RTS, with weakness only being present secondary to pain.^7^ Radial tunnel syndrome is differentiated from posterior interosseus nerve syndrome because of a lack of motor symptoms.^2^^,^^5^ Physical examination maneuvers that elicit pain in patients with RTS are resisted middle finger extension and resisted supination, both of which elicit pain in the muscles overlying the radial head. This is in contrast to the location of pain elicited by these same maneuvers in patients with lateral epicondylitis, which is located over the lateral humeral epicondyle where the ECRB muscle inserts. Furthermore, local anesthetic injection can be used to differentiate RTS from epicondylitis, as injection into the radial tunnel relieves RTS but will not relieve symptoms of epicondylitis, and vice versa for injection into the lateral epicondyle distribution.

Surgical release of the radial tunnel is indicated in cases wherein conservative measures do not improve symptoms. However, because of the less than ideal success rates reported from surgical decompression, a trial of conservative management is highly recommended for all patients suffering from RTS. Splinting, nonsteroidal anti-inflammatory medication, modification of activity, and stretching are the mainstay of conservative therapy. When these measures fail to bring relief to patients for longer than 3 months, surgical intervention is indicated. Surgical decompression has been found to be effective, yet adverse or unsatisfactory results are well documented in the literature, with studies reporting 60% to 70% good to excellent results.^2^^,^^6^ A recent retrospective study found that while 82% of patients who underwent radial tunnel release had relief of their pain, one-third of those continued to have moderate to severe disability affecting their ability to carry out work. Those with the highest satisfaction rates were patients with simple RTS, whereas those with lower satisfaction rates were patients with concurrent nerve compression syndromes, those receiving workman's compensation, or those suffering from lateral epicondylitis.^3^^,^^6^ Several techniques for radial tunnel release have been described, including both posterior (Thompson's) and anterior (Henry's) approaches to the tunnel. Three planes of dissection exist when using the posterior/dorsal approach. The first and most common approach involves division between the ECRB and extensor digitorum communis muscles. Alternative methods include dividing between the brachioradialis and extensor carpi radialis longus muscles or a transmuscular brachioradialis splitting technique. The anterior approach is often preferred when nerve compression exists proximal to the elbow. This allows more complete exploration of the nerve as it crosses the elbow and divides into its distal branches. With these techniques, decompression is achieved by release of the arcade of Frohse, the distal edge of the supinator muscle, or fibrous bands that exist superficial to the radiocapitellar joint.^6^

Radial tunnel syndrome is a rare and poorly understood condition that causes significant burden on afflicted patients. The root mechanism of disease is the entrapment of the deep branch of the radial nerve within the radial tunnel. Diagnosis is arrived at completely based on clinical examination, as radiographic, pathological, and electrodiagnostic evidence is limited. Diagnosis is further complicated by related conditions such as lateral epicondylitis. Surgery is a viable option for those who are refractory to conservative measures, but patients for surgical intervention must be carefully selected to achieve satisfactory results.^3^

## Figures and Tables

**Figure 1:**
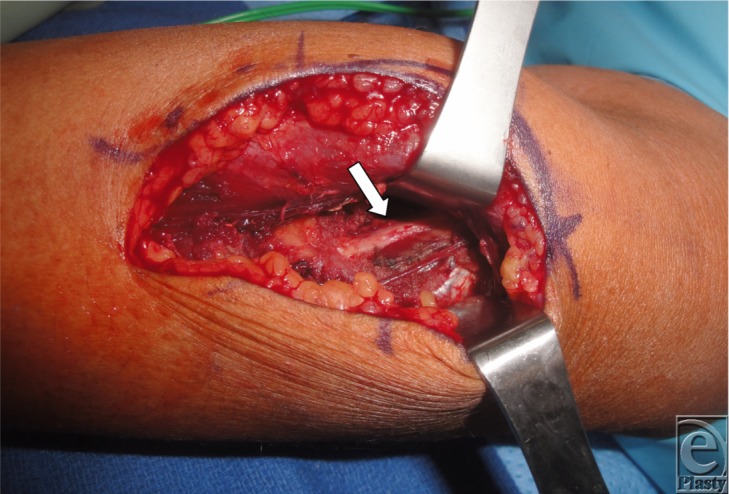
Radial nerve after release.

**Figure 2:**
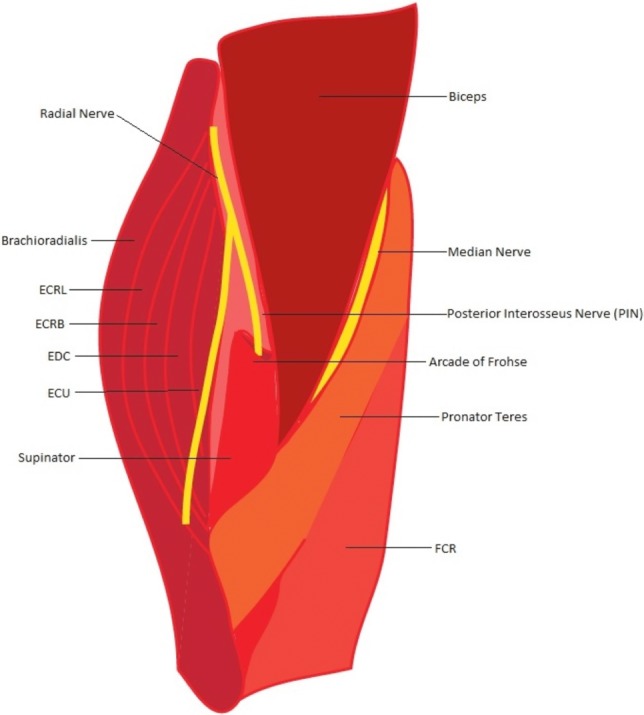
Schematic of radial tunnel anatomy.
